# Supplementary figures and images for: Randomized feasibility trial of the Scleroderma Patient-centered Intervention Network hand exercise program (SPIN-HAND)
Source: PeerJ. 2022 Aug 4;10:e13471. doi: 10.7717/peerj.13471 (PMC9357372; doi:10.7717/peerj.13471)

Figure 7. SPIN Cohort and SPIN-HAND Feasibility Trial Flow

**
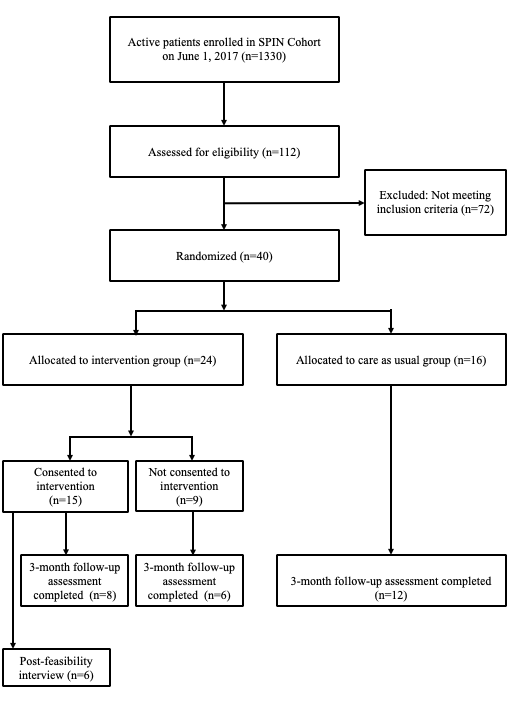
**

Supplement: Supplemental Information 1 [file peerj-10-13471-s001.docx]
