# Supplementary material for: Randomized feasibility trial of the Scleroderma Patient-centered Intervention Network hand exercise program (SPIN-HAND)
Source: PeerJ. 2022 Aug 4;10:e13471. doi: 10.7717/peerj.13471 (PMC9357372; doi:10.7717/peerj.13471)
Supplement: Supplemental Information 2 [file peerj-10-13471-s002.docx]

**APPENDIX A**

**SPIN-HAND INTERVENTION PATIENT INTERVIEWS**

Did you use a computer or tablet or both to access the SPIN-HAND Program?

Can you please tell us about your experience with the SPIN-HAND Program, including things that you liked about the program and things that could be improved?

PROCESS

1. Did the initial invitation email provide you with the information you needed to understand how to sign up for the study?

Yes. No.

If No: What information was missing?

2. Did you find the follow up telephone call you received within 48 hours of the invitation email to be helpful?

Yes. No.

If No: Why not?

PURPOSE

3. Did you understand the objective of the SPIN-HAND program?

Yes. No.

If No: How could the objective be clarified?

4. Did you find the information provided in the SPIN-HAND program relevant?

Yes. No.

If No: How could the information provided be made more relevant for you or other scleroderma patients?

WORDS AND LANGUAGE

5. Did you find that the intervention used common, everyday language that was easy to understand?

Yes. No.

If No: Can you give an example of something or some word(s) that you did not understand?

6. Did you understand all the medical terms or, if not, were they clearly explained in the SPIN-HAND program?

Yes. No.

If No: Can you give an example of medical term(s) that you did not understand?

CONTENT, ORGANIZATION, NAVIGATION

7. Did you find that the SPIN-HAND program is broken down into manageable chunks or sections?

Yes. No.

If No: Which parts of the content weren’t broken down into manageable chunks or sections and how could we improve them?

8. Did you find the different pages or sections of the program to be clearly indicated?

Yes. No.

If No: What section(s) could be more clearly labeled?

9. Did you find it easy to navigate through the intervention and to understand where to go next?

Yes. No.

If No: How could the different steps to navigate the intervention be more clearly explained?

10. Did you consult the “More info” tab (Scleroderma and your hands, FAQ, Patient stories)?

Yes. No.

If No: Why not?

11. Did you experience any technical difficulties while using the intervention?

Yes. No.

If Yes: What type of technical problems? Did you request assistance from the SPIN team? If you did, was the SPIN team able to help you resolve them?

12. Did you use the website tour?

Yes. No.

If Yes: Was it helpful to learn to navigate the website? Why or why not?

13. Did you use the “My bookmarks” feature?

Yes. No.

If Yes: Did you find it helpful for easily navigating to the pages you wanted? Why or why not?

VISUAL AIDS

14. Did the fact that the intervention was introduced by scleroderma experts and patients make the program more relatable?

Why or why not?

15. Did you understand how to correctly perform the exercises from watching the videos and listening to the audio instructions?

Yes. No.

If No: What would have helped you better understand how to correctly perform the exercises?

16. Did you take a look at the “Tips to avoid common mistakes” sections?

Yes. No.

If Yes: Did the pictures of common mistakes and written instructions help you to avoid performing wrong movements? Yes. No.

If No: Why didn’t you use the section on common mistakes section?

17. Were you able to clearly understand the people speaking in the videos?

Yes. No.

If No: Why couldn’t you understand the words in the videos? (e.g. too fast, too soft, mumbling, accent)?; Are there any videos in particular that were more difficult to understand than others? If yes, which one(s);

18. Did you look at the video transcripts?

Yes. No.

If Yes: Were the video transcripts helpful to you? Why or why not?

ACTIONABILITY (Routine, Goal-setting, motivation)

19. Did you set an exercise routine for yourself?

Yes. No.

If Yes: Did you find it easy to set an exercise routine for yourself using the materials in the SPIN-HAND program? Yes. No.

If No: How could the step-by-step approach be improved or better explained?

20. Did you find an exercise routine that fit your ability level and needs?

Yes. No.

If No: What made it hard for you to find an exercise routine that fit your ability level and needs? (e.g., levels not appropriate, time spent on exercises per day or per week not appropriate, other reason)

21. Did you set goals for yourself using the goal setting material?

Yes. No.

Why or why not?

22. Did you incorporate exercises into your planned routine and stick to it?

Yes. No.

If No: What were some obstacles you faced when trying to incorporate the exercises into your routine? How could the SPIN-HAND program have helped you to overcome these obstacles?

23. Did you use the option to share your goals with friends and family via email?

Yes. No.

If Yes: Did the option to share your goals with friends and family via email help you stick to your goals? Yes. No.

If No: What other motivational feature might have been more helpful?

24. Did you set email reminders for yourself?

Yes. No.

If Yes: Did having the option set email reminders for yourself help you incorporate the exercises into your routine? Yes. No.

If No: Did you use another type of reminder to do your exercises?

25. Did you use the feature to track your progress?

Yes. No.

If Yes: Did having the option to track your progress week after week encourage you to continue performing the exercises? Yes. No.

If No: Why not? Did you use any other way to track your progress? Is so, what did you do?

OVERALL APPRECIATION

26. How user-friendly on a 0-10 scale (0, being the worst and 10 being the best possible score) would you rate the SPIN-HAND program?

27. Would you recommend this program to someone with scleroderma?

Yes. No.

If no, why?

28. What grade (on a 0-10 scale, 0 being the worst and 10 being the best possible score) would you give the program?

0 (worst) to 10 (best).

29. Is there anything you want to give us feedback about that was not included in this interview?

| **Measure** | **Intervention N completed** | **Intervention**  **Mean (SD)** | **Controls N completed** | **Controls Mean (SD)** | **Standardised Mean Difference Effect Size (95% confidence interval)** |
| --- | --- | --- | --- | --- | --- |
| PROMIS-29 Anxiety  Baseline  Month 3 | 24  14 | 53.8 (10.5)  48.7 (8.2) | 16  12 | 56.7 (9.9)  53.2 (9.6) | -0.52 (-1.30, 0.27) |
| PROMIS-29 Depression  Baseline  Month 3 | 24  14 | 51.8 (10.1)  47.4 (8.0) | 16  12 | 53.7 (9.9)  51.6 (9.8) | -0.47 (-1.25, 0.31) |
| PROMIS-29 Fatigue  Baseline  Month 3 | 24  14 | 58.3 (12.0)  55.7 (10.7) | 16  12 | 59.3 (7.4)  58.7 (8.9) | -0.30 (-1.07, 0.48) |
| PROMIS-29 Sleep  Baseline  Month 3 | 24  14 | 54.3 (8.7)  50.4 (6.7) | 16  12 | 53.6 (9.6)  54.3 (6.1) | -0.61 (-1.40, 0.18) |
| PROMIS-29 Social roles  Baseline  Month 3 | 24  14 | 46.0 (8.7)  49.5 (10.0) | 16  12 | 42.3 (7.8)  40.7 (8.2) | 0.90 (0.12, 1.75) |
| PROMIS-29 Pain interference  Baseline  Month 3 | 24  14 | 57.8 (9.9)  56.1 (7.8) | 16  12 | 61.7 (5.9)  60.6 (6.7) | -0.62 (-1.41, 0.18) |
| PROMIS-29 Pain intensity  Baseline  Month 3 | 24  14 | 4.7 (2.8)  4.9 (2.7) | 16  12 | 5.9 (1.9)  5.3 (1.9) | -0.13 (-0.91, 0.64) |

**Appendix Table 1. Pre- and Post- Intervention Total Scores for PROMIS-29v2 domains**

**Appendix Table 2. Pre- and Post- Intervention frequencies for EQ-5D-5L dimensions**

| **EQ-5D dimension** |  | **Intervention N completed** | **Intervention**  **N (%)** | **Controls N completed** | **Controls**  **N (%)** |
| --- | --- | --- | --- | --- | --- |
| Mobility  Baseline  Month 3 | Level 1  Level 2  Level 3  Level 4  Level 5  Level 1  Level 2  Level 3  Level 4  Level 5 | 24  24  24  24  24  14  14  14  14  14 | 7 (29.2)  8 (33.3)  8 (33.3)  1 (4.2)  0 (0.0)  5 (35.7)  7 (50.0)  0 (0.0)  2 (14.3)  0 (0.0) | 16  16  16  16  16  12  12  12  12  12 | 5 (31.3)  6 (37.5)  3 (18.8)  2 (12.5)  0 (0.0)  7 (58.3)  1 (8.3)  2 (16.7)  2 (16.7)  0 (0.0) |
| Self-care  Baseline  Month 3 | Level 1  Level 2  Level 3  Level 4  Level 5  Level 1  Level 2  Level 3  Level 4  Level 5 | 24  24  24  24  24  14  14  14  14  14 | 10 (41.7)  9 (37.5)  4 (16.7)  0 (0.0)  1 (4.2)  8 (57.1)  3 (21.4)  2 (14.3)  1 (7.1)  0 (0.0) | 16  16  16  16  16  12  12  12  12  12 | 7 (43.8)  7 (43.8)  1 (6.3)  1 (6.3)  0 (0.0)  4 (33.3)  7 (58.3)  1 (8.3)  0 (0.0)  0 (0.0) |
| Usual Activity  Baseline  Month 3 | Level 1  Level 2  Level 3  Level 4  Level 5  Level 1  Level 2  Level 3  Level 4  Level 5 | 24  24  24  24  24  14  14  14  14  14 | 6 (25.0)  8 (33.3)  8 (33.3)  2 (8.3)  0 (0.0)  4 (28.6)  6 (42.9)  2 (14.3)  2 (14.3)  0 (0.0) | 16  16  16  16  16  12  12  12  12  12 | 0 (0.0)  8 (50.0)  5 (31.3)  3 (18.8)  0 (0.0)  2 (16.7)  5 (41.7)  4 (33.3)  1 (8.3)  0 (0.0) |
| Pain/discomfort  Baseline  Month 3 | Level 1  Level 2  Level 3  Level 4  Level 5  Level 1  Level 2  Level 3  Level 4  Level 5 | 24  24  24  24  24  14  14  14  14  14 | 2 (8.3)  10 (41.7)  9 (37.5)  3 (12.5)  0 (0.0)  1 (7.1)  5 (35.7)  5 (35.7)  3 (21.4)  0 (0.0) | 16  16  16  16  16  12  12  12  12  12 | 1 (6.3)  4 (25.0)  8 (50.0)  3 (18.8)  0 (0.0)  0 (0.0)  2 (16.7)  10 (83.3)  0 (0.0)  0 (0.0) |
| Anxiety/depression  Baseline  Month 3 | Level 1  Level 2  Level 3  Level 4  Level 5  Level 1  Level 2  Level 3  Level 4  Level 5 | 24  24  24  24  24  14  14  14  14  14 | 8 (33.3)  11 (45.8)  3 (12.5)  2 (8.3)  0 (0.0)  11 (78.6)  2 (14.3)  1 (7.1)  0 (0.0)  0 (0.0) | 16  16  16  16  16  12  12  12  12  12 | 5 (31.3)  8 (50.0)  3 (18.8)  0 (0.0)  0 (0.0)  6 (50.0)  4 (33.3)  1 (8.3)  1 (8.3)  0 (0.0) |
| VAS  Baseline, mean (SD)  Month 3, mean (SD)^a^ |  | 24  14 | 64.9 (19.7)  66.9 (19.8) | 16  12 | 56.8 (15.9)  54.9 (13.1) |

^a^Standardized Mean Difference (95% Confidence Interval) = 0.70 (-0.10, 1.49)
