# Supplementary material for: Randomized feasibility trial of the Scleroderma Patient-centered Intervention Network hand exercise program (SPIN-HAND)
Source: PeerJ. 2022 Aug 4;10:e13471. doi: 10.7717/peerj.13471 (PMC9357372; doi:10.7717/peerj.13471)
Supplement: Supplemental Information 5 [file peerj-10-13471-s005.pdf]

Variable Name	Description	Values
Group1	Randomization assignment	0 = Care as usual<br>1 = SPIN-HAND Program
Consent1	Consent to treatment	0 = No<br>1 = Yes
Gender1	Sex	0 = male<br>1 = female
Age1	Age in years	Continuous
Country1	Country of enrollment	1 = Canada<br>2 = United States<br>3 = United Kingdom
Education	Years of education	Continuous
Ethnicity_Combined1	Race/ethnicity	1 = White<br>2 = Black<br>3 = Other
Marital	Marital status	1 = Married<br>2 = Common Law<br>3 = Separated/divorced<br>4 = Widowed<br>5 = Single
Mrss1	Modified Rodnan Skin Score	Continuous
Smalljoints1	Small joints contractures	1 = Negative<br>2 = Positive
Largejoints1	Large joint contractures	1 = Negative<br>2 = Positive
Subtype1	Disease subtype	0 = Limited subtype<br>1 = Diffuse subtype
Tendon1	Tendon friction rubs	0 = Negative<br>1 = Positive
Timediagnosis1	Time since diagnosis in years	Continuous
Timenonray1	Time since onset first non-Raynaud's symptom or sign in years	Continuous
Timeray1	Time since onset Raynaud's in years	Continuous
Cochintotal1	Cochin Hand Function Scale at baseline	Continuous
Cochintotal2	Cochin Hand Function Scale at 3 months	Continuous
Prfuntot1	PROMIS-29 Physical Function T-score at baseline	Continuous
Prfuntot2	PROMIS-29 Physical Function T-score at 3 months	Continuous
Prfatiguetot1	PROMIS-29 Fatigue T-score at baseline	Continuous
Prfatiguetot2	PROMIS-29 Fatigue T-score at 3 months	Continuous

Prdeptot1	PROMIS-29 Depression T-score at baseline	Continuous
Prdeptot2	PROMIS-29 Depression T-score at 3 months	Continuous
Pranxtot1	PROMIS-29 Anxiety T-score at baseline	Continuous
Pranxtot2	PROMIS-29 Anxiety T-score at 3 months	Continuous
Prpaintot1	PROMIS-29 Pain Interference T-score at baseline	Continuous
Prpaintot2	PROMIS-29 Pain Interference T-score at 3 months	Continuous
Prrolestot1	PROMIS-29 Social Roles T-score at baseline	Continuous
Prrolestot2	PROMIS-29 Social Roles T-score at 3 months	Continuous
Prsleeptot1	PROMIS-29 Sleep T-score at baseline	Continuous
Prsleeptot2	PROMIS-29 Sleep T-score at 3 months	Continuous
Promis291	PROMIS-29 Pain intensity at baseline	Continuous
Promis292	PROMIS-29 Pain intensity at 3 months	Continuous
Mobility1	EQ-5D Mobility at baseline	1 = level 1 (no problems)<br>2 = level 2 (slight problems)<br>3 = level 3 (moderate problems)<br>4 = level 4 (severe problems)<br>5 = level 5 (extreme problems)
Mobility2	EQ-5D Mobility at 3 months	1 = level 1 (no problems)<br>2 = level 2 (slight problems)<br>3 = level 3 (moderate problems)<br>4 = level 4 (severe problems)<br>5 = level 5 (extreme problems)
Selfcare1	EQ-5D Self-care at baseline	1 = level 1 (no problems)<br>2 = level 2 (slight problems)<br>3 = level 3 (moderate problems)<br>4 = level 4 (severe problems)<br>5 = level 5 (extreme problems)
Selfcare2	EQ-5D Self-care at 3 months	1 = level 1 (no problems)<br>2 = level 2 (slight problems)<br>3 = level 3 (moderate problems)<br>4 = level 4 (severe problems)<br>5 = level 5 (extreme problems)
Activities1	EQ-5D Usual Activity at baseline	1 = level 1 (no problems)<br>2 = level 2 (slight problems)<br>3 = level 3 (moderate problems)<br>4 = level 4 (severe problems)

		5 = level 5 (extreme problems)
Activities2	EQ-5D Usual Activity at 3 months	1 = level 1 (no problems)<br>2 = level 2 (slight problems)<br>3 = level 3 (moderate problems)<br>4 = level 4 (severe problems)<br>5 = level 5 (extreme problems)
Pain1	EQ-5D Pain/discomfort at baseline	1 = level 1 (no problems)<br>2 = level 2 (slight problems)<br>3 = level 3 (moderate problems)<br>4 = level 4 (severe problems)<br>5 = level 5 (extreme problems)
Pain2	EQ-5D Pain/discomfort at 3 months	1 = level 1 (no problems)<br>2 = level 2 (slight problems)<br>3 = level 3 (moderate problems)<br>4 = level 4 (severe problems)<br>5 = level 5 (extreme problems)
Anxiety1	EQ-5D Anxiety/depression at baseline	1 = level 1 (no problems)<br>2 = level 2 (slight problems)<br>3 = level 3 (moderate problems)<br>4 = level 4 (severe problems)<br>5 = level 5 (extreme problems)
Anxiety2	EQ-5D Anxiety/depression at 3 months	1 = level 1 (no problems)<br>2 = level 2 (slight problems)<br>3 = level 3 (moderate problems)<br>4 = level 4 (severe problems)<br>5 = level 5 (extreme problems)
Health1	EQ-5D visual analogue scale at baseline	Continuous
Health2	EQ-5D visual analogue scale at 3 months	Continuous
