# Supplementary material for: Randomized feasibility trial of the Scleroderma Patient-centered Intervention Network hand exercise program (SPIN-HAND)
Source: PeerJ. 2022 Aug 4;10:e13471. doi: 10.7717/peerj.13471 (PMC9357372; doi:10.7717/peerj.13471)
Supplement: Supplemental Information 6 [file peerj-10-13471-s006.pdf]

PROTOCOL TITLE:

Feasibility Trial of an Internet-based Exercise Program to Improve Hand Function in Patients with Scleroderma: A Scleroderma Patient-centered Intervention Network (SPIN) Study

1. Introduction

Canadian and international rare disease plans emphasize the need for accessible disease-management tools to complement basic medical care.[1,2] However, the small number of patients with any rare disease is a significant barrier to testing interventions. Systemic sclerosis (SSc, or scleroderma) is a rare autoimmune disease characterized by thickening and fibrosis of the skin and internal organ involvement.[3,4] Skin and tendon involvement of the hands with significant functional impairment is nearly universal.[5-8] Members of our research team recently showed that a supervised multifaceted home-based exercise program for SSc, which included hand exercises, reduced disability and improved hand function.[9] The program, however, was short-term, did not focus specifically on improving hand function, and the benefits were not sustained. Furthermore, most SSc treatment centres do not have the expertise or resources to provide such a program, and most SSc patients are not cared for in specialty centres, which are located in major metropolitan areas.

Given the challenges of conducting high-quality, adequately powered clinical trials and disseminating disease management tools in a rare disease context, proven self-management and rehabilitation interventions are typically not available to rare disease patients.[10-15] To address this gap, the Scleroderma Patient-centered Intervention Network (SPIN)[10,11] was funded by a CIHR Team Grant to (1) assemble a large, multinational cohort of SSc patients as a framework for large-scale rehabilitation and self-management intervention trials; and (2) to develop, test, and disseminate effective disease management tools to patients with SSc. SPIN uses a novel cohort-based trial design, in which patient outcome data are collected at regular 3-month intervals and trials are embedded in the Cohort.[10] The SPIN Cohort (*JGH REC Protocol #12-123*) has currently enrolled >1400 patients from 31 centres and anticipates growing to 2000 patients by the end of 2016.

The SPIN Hand Exercise Trial (SPIN-HAND) is a pragmatic RCT embedded in the SPIN Cohort that will evaluate the effect on hand function and health-related quality of life (HRQL) of offering SPIN's online hand exercise program, in addition to usual care, to Cohort patients who have at least mild hand function limitations (JGH REC Protocol #12-123a). Consistent with current best practice recommendations for intervention development and testing, prior to full-scale RCTs, feasibility of the intervention and trial methods will be assessed to ensure that trial methodology is robust, feasible, and consistent with patient expectations.[16-18] The present proposal is requesting ethics approval for conducting feasibility testing of the SPIN-HAND program in the SPIN Cohort.

The SPIN-HAND exercise program was designed by patients and experts in rehabilitation, behavioural therapies, and e-health interventions. The program core consists of 4 modules that address specific aspects of hand function, including Thumb Flexibility and Strength; Finger Bending; Finger Extension; and Wrist Flexibility and Strength. The program also integrates tools to support key components of successful self-management programs, including goal-setting and feedback, social modeling, and mastery experiences.[19-22] SPIN Cohort patients with at least mild hand function limitations and an

indicated interest in using an online hand exercise program will be randomized to be offered the hand exercise program or usual care only.

SPIN has received a 5-year Emerging Team Grant for Rare Diseases from the Canadian Institutes of Health Research (CIHR) to fund establishment of the SPIN Cohort (201108TR3-267681-TRF-CFAF-165164; Principal Investigator, Brett D. Thombs; 2012-2017, $1,499,765) and the feasibility trial for which we are currently seeking ethics approval. In addition to CIHR funding, SPIN has received institutional contributions of $75,000 from the Lady Davis Institute for Medical Research of the Jewish General Hospital for database development and $125,000 from McGill University for student training. Prior to that, SPIN was awarded a total of $45,500 from the Scleroderma Society of Canada, the Scleroderma Society of Ontario, and Sclérodermie Québec, for development work toward this project. SPIN was recently awarded funds from The Arthritis Society (SOG-16-380; Principal Investigator, Brett D. Thombs; 2016-2019, $359,686) to conduct a full-scale RCT of the SPIN-HAND program in the Cohort, as well as CIHR-bridge funding (201604PJT-364343; Principal Investigator, Brett D. Thombs; 2016-2017, $100,000) to support the SPIN Cohort framework.

2. Background and Rationale

Rare diseases are chronic, disabling and often life threatening medical conditions. Individually, rare diseases affect fewer than 1 in 2000 people, but altogether, more than 1 in 15 people (6-8%) have a rare disease.[23-25] The burden and impact on HRQL of most rare diseases is high.[26] However, many patients struggle to get appropriate medical care and supportive services to cope with the emotional and social consequences of their disease.[12-15] Psychosocial, educational and rehabilitation interventions could contribute to alleviating distress and improve function, but there is not evidence of effectiveness of these approaches for rare diseases.[10] These interventions are an important component of patient-centered care in more common diseases, but there are barriers to developing, testing and disseminating these programs in rare diseases.[10] This is an important care gap because many rare disease patients face unique challenges that are often not addressed by interventions developed for more common conditions.[12-15]

SSc is a rare autoimmune disease that affects the skin and internal organs including the lungs, gastrointestinal tract and cardiovascular system.[3,4] SSc is notable for a wide range of patient reported problems, including limitations in physical mobility and hand function, pain, fatigue, sleep disturbance, depression, sexual dysfunction, and changes in appearance.[7,27-29] There are no disease-modifying treatments for SSc and its management is predicated on identifying organ-specific disease manifestations and initiating targeted therapies.[30] Because there is no cure, a primary goal of care is to reduce symptoms and disability, and to improve HRQL. As with many rare diseases, however, most patients with SSc do not have access to educational, psychological and rehabilitation programs, despite the high need that has been reported by this group.[27,30] Evidence-based educational, psychological and rehabilitation interventions to meet the specific needs of patients with SSc are not currently available.

SPIN was established to address this issue.[10] SPIN is a collaboration of key scleroderma research centers, clinicians, patient organizations and investigators from 8 countries. SPIN is in the process of enrolling 1,500-2,000 SSc patients for an ongoing web-based cohort dedicated to better understanding problems important to scleroderma patients, validating outcome measures, and informing development of interventions. SPIN teams are also developing a series of online interventions, including (i) general

SSc self-management, (ii) support for better coping with emotional distress; (iii) support for managing body image distress; and (iv) hand exercises, which will be tested through the Cohort framework.

The proposed SPIN-HAND feasibility trial is a pragmatic RCT embedded in the SPIN Cohort that will evaluate the feasibility of conducting full-scale RCT on the SPIN-HAND exercise program. We will randomize 36-40 SPIN Cohort patients with at least mild hand function limitations and an indicated interest in using an online hand exercise program to be offered the hand exercise program or usual care only.

3. Objectives

To examine the feasibility of the SPIN-HAND trial, by obtaining data related to the study's process, required resources and management, and scientific aspects. This feasibility study will inform us on any changes that need to be implemented before conducting a full-scale RCT of the SPIN-HAND program.

4. Setting, Sample, and Recruitment

The SPIN Cohort and Study Design: The SPIN-HAND feasibility study is a small randomized pilot study embedded in the SPIN Cohort. SPIN Cohort patients must have a SSc diagnosis based on 2013 ACR/EULAR criteria confirmed by a SPIN physician, be ≥18 years old, be able to give informed consent, and be fluent in English or French. Patients not able to access or respond to questionnaires via the internet are excluded. Eligible patients are recruited at SPIN sites during regular medical visits, and written informed consent is obtained. A medical data form is submitted online by the site to enrol patients. Cohort patients complete outcome measures via the internet upon enrolment and subsequently every 3 months. The SPIN Cohort was approved by the Research Ethics Committee of the Jewish General Hospital, Montreal (JGH REC Protocol #12-123), and by ethics committees of each recruiting site. Currently, 31 sites in Canada, the USA, France and the UK recruit patients.

The SPIN Cohort is designed to conduct trials using an embedded cohort multiple RCT design, which has been approved by the JGH REC (Protocol #12-123). In the cmRCT design,[38] patients enrol in an observational cohort with regular outcome measurement. Patients consent to (1) allow their data to be used for observational studies; (2) allow their data to be used to assess intervention trial eligibility and, if eligible, be randomized; and (3) if eligible and randomized to usual care, use their data to evaluate intervention effectiveness without being notified that they have been randomized to the usual care group and not offered the intervention. As part of the enrolment in the SPIN Cohort, patients consented to be contacted by SPIN personnel to be invited to participate in SPIN interventions.

Participants: In total, 32-40 English-speaking SPIN Cohort participants will be enrolled in the SPIN-HAND feasibility study, of whom 16-20 will be offered to use the SPIN-HAND program. Of the patients currently enrolled in the SPIN Cohort, 74 (6%) are French-speaking patients from Quebec. Patients who are offered the SPIN-HAND intervention as part of the present feasibility study will be excluded from the full-scale RCT (JGH protocol #12-123a). In order to ensure that there will be an adequate number of French-speaking patients to be included in the full-scale RCT, only English-speaking patients will be included in the SPIN-HAND feasibility trial. Thus, for the feasibility trial, eligible patients will be able to use the online intervention in English, have at least mild hand function

limitations (Cochin Hand Function Scale[31] (CHFS) ≥ 3) and have indicated high interest in using an online hand exercise intervention (≥7 on 0-10 scale).

Randomization, Allocation Concealment, and Consent: Randomization to be offered versus not offered the exercise program will occur at the time of patients' regular SPIN Cohort assessments. Eligible patients, based on questionnaire responses, will be randomized automatically using a feature in the SPIN Cohort platform, which provides immediate randomization and complete allocation sequence concealment. Patients randomized to be offered the intervention will receive an automated email invitation including a link to the SPIN-HAND program and the SPIN-HAND feasibility study informed consent form. At initial login, patients will be prompted to provide written consent to participate in the SPIN-HAND feasibility study by verifying agreement with each consent element and providing their own email address as the signature. The patient will then be automatically re-directed to the introduction page of the SPIN hand exercise program. If a patient logs out before agreeing to the terms of the consent form, upon subsequent logins the consent form will be displayed until the patient provides consent. Contact information of SPIN personnel will be available to answer any questions patients may have. SPIN personnel will also contact patients by phone, usually within 48 hours of sending the invitation email, to describe the study, review the consent form, and answer questions. Patients who accept the offer can use the web link to enter the secure intervention site login. Email and phone technical support will be available to help patients with the consent process and to access and use the intervention site.

Blinding: In most pragmatic trials, patients are not blinded to intervention status and possible patient biases are accepted as part of the response to being offered a treatment as may occur in practice.[35,36] Disappointment bias, however, can occur in conventional trial designs when a patient enrolls in a trial to receive an intervention, but is allocated to usual care.[32-35] For this reason, in the cmRCT design,[35] patients who are not offered an intervention are not notified that they have not been offered the intervention. This replicates actual practice, where patients are not typically advised about treatments that are not options, and reduces risk of disappointment bias.[32,33,35] All patients in the SPIN Cohort are aware that SPIN will conduct intervention trials and are routinely asked about potential interest in 8-10 possible interventions, but are not informed that any particular intervention may be available unless they are offered to try the intervention.

5. Intervention and Comparator

Home-based exercise rehabilitation programs have been shown to improve hand function in SSc[9] and rheumatoid arthritis.[36] SPIN's hand exercise program is based on these programs and integrates key components of successful disease self-management programs,[36-38] including goal-setting and feedback, social modeling, and mastery experiences.[19,39]

The SPIN-HAND exercise program was designed by SPIN experts in physical medicine, rehabilitation, physical and occupational therapy, and behavioural therapies, together with patient representatives. The core of the program consists of 4 modules that address specific aspects of hand function, including (1) Thumb Flexibility and Strength (3 exercises); (2) Finger Bending (3 exercises); (3) Finger Extension (3 exercises); and (4) Wrist Flexibility and Strength (2 exercises). Patients can select the modules in the order that they prefer, based on a description of the type of function that the module is targeting to maintain or improve. The program includes sections on developing a personalized program, goal-setting strategies and examples, progress tracking, sharing goals and progress with friends and family, and

patient stories of experiences with hand disability and hand exercises. The program utilizes an engaging and easy to navigate web interface. Instructional videos with SSc patients demonstrate and explain how to perform each exercise properly with additional pictures to illustrate common mistakes. Separate versions of each exercise are available for patients with mild/moderate and more severe hand involvement. Some exercise videos that are targeted to patients with severe hand involvement are complemented with pictures to illustrate alternate versions on how to perform the exercise.

Patients are provided guidance on selecting intervention intensity levels. For the first 4 weeks of the program, patients focus on exercises in one module per week and are encouraged to do the exercises 3-5 times per week. Time per day graduates from 3-4 minutes in week 1 to 5-15 minutes in week 4. Starting with week 5, patients can select from a menu of program options that fit their needs and schedule. These range from 5-10 minutes per day to 30-35 minutes per day.

6. Outcomes

The aim of the SPIN-HAND feasibility study is to collect data related to the study's *process*, in order to assess the feasibility of the steps that need to take place as part of the main study; *required resources and management* (e.g., personnel and data management issues); and *scientific aspects* (outcome assessments). Data will be used to determine whether it is feasible to carry on the main study or whether changes need to be made before conducting a full-scale RCT of the SPIN-HAND program. The SPIN-HAND feasibility study is not meant for hypothesis testing or effect size estimation, as the sample size is not appropriate to do so. The feasibility trial outcomes related to the process and resources will be assessed throughout the duration of the feasibility trial, and patient feedback will be obtained at 3 months post-randomization.

The collected measures of feasibility include:

- How many patients in the SPIN Cohort meet the cut-off thresholds for eligibility (provides an estimation of how long recruitment for the full-scale RCT will take);
- How well the enrolment and randomization procedure work;
- The percentage of patients who accept the offer of the intervention and consent to participation;
- Completeness of online data collection for each arm at baseline and at 3 months (as a percentage of patients randomized);
- Completeness of the automatic usage log data values collected (quality of data collected or if any important data were not collected);
- Assess that data coming from the SPIN Cohort and SPIN-HAND platforms can be successfully linked;
- Variability in outcome measures and completion rates of outcome variables;
- Personnel requirements to call enrolled patients and help them with accessing the SPIN-HAND program;
- Challenges for study personnel;
- Technological performance of the online SPIN-HAND program;

User feedback on the intervention:

- Usage of the SPIN-HAND program for patients randomized in the intervention arm will be explored by examining objective intervention usage data collected by the SPIN-HAND program. These data will provide detailed information on number of logins, number of modules accessed,

goals set, as well as time spent on each webpage.

- Patient interviews will be conducted 3-months post-randomization to assess the accessibility of the intervention, barriers to participating, and user feedback, and to explore patients' experience of taking part in the trial including difficulties, positive elements and other salient issues. Data collection will be guided by the PEMAT questionnaire (See Appendix A).

Outcome Measures from the SPIN Cohort Assessments:

The objectives of the SPIN-HAND full-scale RCT will be to evaluate the effect of being offered access to SPIN's online hand exercise program, in addition to usual care, on hand function, functional health outcomes, and HRQL for SSc patients with at least mild hand function limitations, compared to usual care alone. In both the present feasibility study and the full-scale RCT, outcome measures that are routinely assessed as part of the SPIN Cohort assessments every 3-months will be evaluated. For the present feasibility study, we will assess variability in outcome measures and completion rates of outcome variables.

- The Cochin Hand Function Scale (CHFS) was developed to measure functional ability of the hand among patients with rheumatic diseases and has been validated and used extensively in SSc. The 18-item CHFS[28] measures ability to perform daily hand-related activities (e.g., kitchen, dressing oneself, hygiene, writing/typing). Items are scored on a 0-5 Likert scale (0=*without difficulty;* 5=*impossible*). Higher scores indicate less functionality. The total score is obtained by adding the scores of all items (range 0-90). The CHFS has good convergent validity with general functional disability measures and good sensitivity to change.[5,31,40,41] It has been validated in SSc.[41]
- Patient-reported health status will be measured using the 29-item Patient Reported Outcomes Measurement Information System (PROMIS-29) profile version 2.0. The PROMIS-29 measures 8 domains of health status with 4 items for each of 7 domains (physical function, anxiety, depression, fatigue, sleep disturbance, social roles and activities, pain interference) plus a single item for pain intensity. Items are scored on a 5-point scale (range 1-5), with different response options for different domains, and the single pain intensity item is measured on an 11-point rating scale. Higher scores represent more of the domain being measured; that is, better physical function and ability to participate in social roles and activities, but higher levels of anxiety, depression, fatigue, sleep disturbance, pain interference, and pain intensity. Total raw scores are obtained by summing item scores for each domain, which are converted into T-scores standardized from the general US population (mean=50, SD=10). The PROMIS-29 version 2.0 has been validated in SSc.[42,43]
- HRQL will be assessed with the EQ-5D, a 5-item standardized questionnaire, measuring 5 dimensions (mobility, self-care, usual activities, pain/discomfort, and anxiety/depression). The items are rated from 1 (no problems) to 3 (extreme problems). Scores range from -0.59 to 1.00 and reflect overall HRQL.[44]

Harms/Adverse events:
The risk of adverse events occurring as a consequence of the SPIN-HAND program is very low. All hand exercises recommended are explained in detail with an emphasis on choosing a level that is comfortable for the patient. Nonetheless, adverse events will be recorded. Where necessary, the event

will be discussed with clinical members of team and a referral to local physicians will be made. Serious adverse events that do occur will also be reported to the ethics committee.

Sample size:
Guidance on appropriate sample size for feasibility trials varies substantially in the published literature. Published guidelines[45] suggest that at least 9% of the estimated sample size for the full-scale RCT should be included in the feasibility study. With an estimated effect size of 0.3, for 80% power with $\alpha = 0.05$, we will require data from ≥ 352 patients in the full-scale RCT. Assuming 20% loss to follow-up, which exceeds current missing data rates in the SPIN Cohort substantially, we would need to randomize 440 patients. Thus, we will include between 32 (9% of 352) and 40 (9% of 440) patients for this feasibility trial, approximately 16 to 20 patients allocated to each of the two arms.

Statistical analyses:
A description of feasibility outcomes will be presented, including patient eligibility and recruitment, and numbers and percentages of patients who do not respond to follow-up measures. Use of the internet intervention will be described by presenting the frequency of logins and time spent on the SPIN-HAND program. Analysis of outcome measures will include the completeness of data and presence of floor or ceiling effects. Descriptive statistics will be used to provide means and standard deviations for the measures. Qualitative information and information related to management and usability of the SPIN-HAND program will inform any necessary changes to the intervention or trial procedures.

The study will be conducted according to the principles of the *Tri-Council Policy Statement: Ethical Conduct for Research Involving Humans* (2010). Participants can leave the study at any time for any reason if they wish to do so without any consequences. The SPIN investigators will inform the participants and the research ethics board if anything occurs, on the basis of which it appears that the disadvantages of participation may be significantly greater than was foreseen in the research proposal. In that case, the study would be suspended pending further review by the research ethics board, except insofar as suspension would jeopardise the participants' health.

Outcome measures are completed through the participants' regular SPIN Cohort assessments. The SPIN Cohort uses a secure electronic data management platform designed and managed by the *Information Management Services (IMS) of the Centre for Clinical Epidemiology, Lady Davis Institute for Medical Research*, to which only Dr. Thombs or a SPIN team member at the Jewish General Hospital authorized by Dr. Thombs has access. To protect the participants' privacy, upon inclusion in the SPIN Cohort, a unique patient identification number has automatically been assigned to each participant. For details on SPIN Cohort platform security, please refer to the ethics documents for the SPIN Cohort (*JGH REC Protocol #12-123*).

Separate from the SPIN Cohort portal, an encrypted database will be created for the SPIN-HAND program, which includes the patient identification number of the patients, and their usage log information. Only requests authorized by the principal investigator (Dr. Brett Thombs) will be granted access to this encrypted information. Data will be kept for ten years after ending the study for ethical and scientific reasons.

The IMS is located in the Center of Clinical Epidemiology, Lady Davis Institute which is only accessible to authorized personnel through an access card system. The server room has an independent

access card system, which is strictly limited to authorized IT personnel. Multiple systems are implemented to protect the data center: temperature control; fire/smoke detection; power outage protection; onsite security for break-ins. The architecture of the servers includes all the programs and components necessary to control the access, the confidentiality and the integrity of the data located on them. The Data Center has all the required and up-to-date security, including: firewall, IDS/IPS, antivirus, anti-spyware, encrypted hard drives, password, audit trail, and secured backups.

The SPIN Data Access and Publication Policy (see *JGH REC Protocol #12-123*) describes in detail how access to data can be obtained by SPIN investigators. In brief, SPIN investigators will be able to obtain SPIN research data only upon approval of a proposal by the *SPIN Data Access and Publication Committee*. Only those data will be shared that are needed to conduct the study. All patient identifiers will be removed prior to sharing data with SPIN investigators. Before approval on the submission of any manuscript, the principal investigator (Dr. Brett Thombs) will make sure that published results are not traceable to individual patients. Data will be sent to SPIN investigators using an encrypted database, and the password for this database will be provided in a separate e-mail to the SPIN investigator.

9. Risks and potential benefits:

We do not anticipate any major safety concerns with the use of the SPIN-HAND program. In addition to patient's participation in the SPIN Cohort, for which they complete outcome measures at regular 3-month intervals, participation in the SPIN-HAND feasibility study will involve having access to an online intervention that teaches hand exercises. The only risks of participation in the SPIN-HAND feasibility study may be some light discomfort during the exercises, especially if participants have not stretched their hands for a while.

Although it is hypothesized that SPIN-HAND will improve hand function in patients with SSc compared to care as usual, it cannot be guaranteed that patients will receive any benefits from this study. However, information learned from this research may lead to better interventions to target important rehabilitation problems for people living with scleroderma, which may benefit other patients in the future. There will be no financial compensation for patients who are participating in the SPIN-HAND feasibility study.

REFERENCES

1. Now is the time: A strategy for rare diseases in a strategy for all Canadians. Canada's rare disease strategy. www.raredisorders.ca/content/uploads/CORD_Canada_RD_Strategy_22May15.pdf. Accessed April 14, 2016.
2. National plans or strategies for rare diseases. www.ec.europa.eu/health/rare_diseases/national_plans/detailed/index_en.htm (Accessed April 14, 2016
3. Seibold J. Scleroderma. In Harris ED, Budd RC, Firestein GS, et al. (Eds.). *Kelley's Textbook of Rheumatology.* Philadelphia, Elsevier, 2005:1279-308.
4. Wigley FM, Hummers LK. Clinical features of systemic sclerosis. In Hochberg MC, Silman AJ, Smolen JS, et al. (Eds.): *Rheumatology.* Philadelphia, Mosby, 2003:1463-80.
5. Rannou F, Poiraudeau S, Berezne A, et al. Assessing disability and quality of life in systemic sclerosis: construct validities of the Cochin Hand Function Scale, Health Assessment Questionnaire (HAQ), Systemic Sclerosis HAQ, and Medical Outcomes Study 36-Item Short Form Health Survey. *Arthritis Rheum*. 2007;57: 94-102.
6. Kallen MA, Mayes MD, Kriseman YL, et al. The Symptom Burden Index: development and initial findings from use with patients with systemic sclerosis. *J Rheumatol*. 2010;37:1692.
7. Bassel M, Hudson M, Taillefer SS, et al. Frequency and impact of symptoms experienced by patients with systemic sclerosis: results from a Canadian National Survey. *Rheumatology*. 2011;50:762-7.
8. Poole JL. Musculoskeletal rehabilitation in the person with scleroderma. *Curr Opin Rheumatol*. 2010;22:205-12
9. Rannou F. Effect of rehabilitation in systemic sclerosis. *Ann Phys Rehabil Med*. 2015;58:e120 (Abstract; full trial report under review; NCT00318188; PI Poiraudeau).
10. Kwakkenbos L, Jewett LR, Baron M, et al. The Scleroderma Patient-centered Intervention Network (SPIN) Cohort: protocol for a cohort multiple randomised controlled trial (cmRCT) design to support trials of psychosocial and rehabilitation interventions in a rare disease context. *BMJ Open.* 2013;3:e003563.
11. Thombs BD, Jewett LR, Assassi S, et al. New directions for patient-centered care in scleroderma: The Scleroderma Patient-centered Intervention Network (SPIN). *Clin Exp Rheumatol*. 2012;30:S23-S29.
12. Kole A, Faurisson F. The voice of 12,000 patients: experiences and expectations of rare disease patients on diagnosis and care in Europe. www.eurordis.org/IMG/pdf/voice_12000_patients/EURORDISCARE_FULLBOOKr.pdf]. Accessed April 14, 2016.
13. Huyard C. What, if anything, is specific about having a rare disorder? Patients' judgements on being ill and being rare. *Health Expect.* 2009;12:361–70.
14. Nettleton S, Watt I, O'Malley L, et al. Understanding the narratives of people who live with medically unexplained illness. *Patient Educ Couns.* 2005;56:205–21.
15. Reimann A, Bend J, Dembski B. [Patient-centred care in rare diseases. A patient organisations' perspective]. *Bundesgesundheitsblatt Gesundheitsforschung Gesundheitsschutz.* 2007;50:1484-93.

16. Kraemer HC, Mintz J, Noda A, Tinklenberg J, Yesavage JA. Caution regarding the use of pilot studies to guide power calculations for study proposals. *Arch Gen Psychiatry*. 2006;63(5):484-489.
17. Thabane L, Ma J, Chu R, et al. A tutorial on pilot studies: The what, why and how. *BMC Med Res Methodol*. 2010;10:1.
18. Van Teijlingen ER, Rennie AM, Hundley V, Graham W. The importance of conducting and reporting pilot studies: The example of the Scottish Births Survey. *J Adv Nurs*. 2001;34(3):289-295.
19. Lorig KR, Holman H. Self-management education: history, definition, outcomes, and mechanisms. *Ann Behav Med.* 2003;26:1-7.
20. Holman H, Lorig K. Perceived self-efficacy in self-management of chronic disease. In: Schwarzer C, editor. *Self-efficacy: Thought control of action*. Pennsylvania: Taylor & Francis, 1992.
21. Marks R, Allegrante JP, Lorig K. A review and synthesis of research evidence for self-efficacy-enhancing interventions for reducing chronic disability: implications for health education practice (part I). *Health Promot Pract.* 2005;6:37-43.
22. Marks R, Allegrante JP, Lorig K. A review and synthesis of research evidence for self-efficacy-enhancing interventions for reducing chronic disability: implications for health education practice (part II). *Health Promot Pract.* 2005;6:148-56.
23. Canadian Organization for Rare Disorders. https://www.raredisorders.ca/about-cord/ Accessed April 14, 2016.
24. Wastfelt M, Fadeel B, Henter JI. A journey of hope: Lessons learned from studies on rare diseases and orphan drugs. *J Intern Med*. 2006;260:1-10.
25. EURORDIS Rare Diseases Europe. www.eurordis.org/content/what-rare-disease. Accessed April 14, 2016.
26. Cohen JS, Biesecker BB. Quality of life in rare genetic conditions: a systematic review of the literature. *Am J Med Genet A.* 2010;152:1136–56.
27. Thombs BD, van Lankveld W, Bassel M, et al. Psychological health and well-being in systemic sclerosis: State of the science and consensus research agenda. *Arthritis Care Res (Hoboken)* 2010;62:1181-9.
28. Kwakkenbos L, Delisle VC, Fox RS, Gholizadeh S, Jewett LR, Levis B, Milette K, Mills SD, Malcarne VL, Thombs BD. Psychosocial aspects of scleroderma. *Rheum Dis Clin North Am* 2015;41:519-28.
29. Jewett LR, Haythornthwaite JA, Thombs BD. Psychosocial issues and care for patients with systemic sclerosis. In Varga J, Denton CP, Wigley FM, eds. Scleroderma: From Pathogenesis to Comprehensive Management. New York: Springer 2012:641-8.
30. Kowal-Bielecka O, Landewe R, Avouac J, et al. EULAR recommendations for the treatment of systemic sclerosis: a report from the EULAR Scleroderma Trials and Research group (EUSTAR). *Ann Rheum Dis* 2009;68:620-8.
31. Duruöz MT, Poiraudeau S, Fermanian J, et al. Development and validation of a rheumatoid hand functional disability scale that assesses functional handicap. *J Rheumatol*. 1996;23:1167-72.
32. Roland M, Torgerson DJ. Understanding controlled trials: What are pragmatic trials? *BMJ*. 1998;316:285.
33. Torgerson DJ, Torgerson CJ. *Designing randomised trials in health, education, and the social sciences: an introduction:* Palgrave Macmillan;2008.

34. Zwarenstein M, Treweek S, Gagnier JJ, et al. Improving the reporting of pragmatic trials: an extension of the CONSORT statement. *BMJ* 2008;337:a2390.
35. Relton C, Torgerson D, O'Cathain A, et al. Rethinking pragmatic randomised controlled trials: introducing the "cohort multiple randomised controlled trial" design. *BMJ*. 2010;340:c1066.
36. Lamb SE, Williamson EM, Heine PJ, et al. Exercises to improve function of the rheumatoid hand (SARAH): a randomised controlled trial. *Lancet*. 2015;385:421-9.
37. Lorig KR, Ritter PL, Laurent DD, et al. The internet-based arthritis self-management program: a one-year randomized trial for patients with arthritis or fibromyalgia. *Arthritis Rheum*. 2008;15:1009-17.
38. Foster G, Taylor SJ, Eldridge SE, et al. Self-management education programmes by lay leaders for people with chronic conditions. *Cochrane Database Syst Rev*. 2007:CD005108.
39. Bandura A. Self-efficacy: toward a unifying theory of behavioral change. Psychol *Rev* 1977;84:191-215., and mechanisms. *Ann Behav Med*. 2003;26:1-7.
40. Poiraudeau S, Chevalier X, Conrozier T, et al. Reliability, validity, and sensitivity to change of the Cochin Hand Function Disability Scale in hand osteoarthritis. *Osteoarthritis Cartilage*. 2001;9:570-577
41. Brower LM, Poole JL. Reliability and validity of the Duruoz Hand Index in persons with systemic sclerosis (scleroderma). *Arthritis Rheum*. 2004;51:805–9.
42. Hinchcliff M, Beaumont JL, Thavarajah K, et al. Validity of two new patient-reported outcome measures in systemic sclerosis: Patient-Reported Outcomes Measurement Information System 29-item health profile and Functional Assessment of Chronic Illness Therapy–Dyspnea Short Form. *Arthritis Care Res*. 2011;63:1620-8.
43. Kwakkenbos L, Thombs BD, Khanna D, Carrier ME, Baron M, Furst D, Gottesman K, van den Hoogen FHJ, Malcarne VL, Mayes MD, Mouthon L, Nielson WR, Poiraudeau S, Riggs R, Sauvé M, Wigley F, Hudson M, Bartlett SJ, SPIN investigators. Validation of the Patient-Reported Outcomes Measurement Information System-29 (PROMIS-29) in Scleroderma and Associations with Clinical Characteristics: A Scleroderma Patient-centered Intervention Network (SPIN) Cohort Study. *Submitted for publication.*
44. Rabin R, de Charro F. EQ-5D: a measure of health status from the EuroQol Group. *Ann Med* 2001;33:337-43.
45. Cocks K, Torgerson DJ. Sample size calculations for pilot randomized trials: a confidence interval approach. J Clin Epidemiol. 2013 Feb;66(2):197-201.

APPENDIX A

SPIN-HAND INTERVENTION PATIENT INTERVIEWS

PROCESS

1. Did the initial invitation email provide you with the information you needed to understand how to sign up for the study?
Yes. No.
If No: What information was missing?

2. Did you find the follow up telephone call you received within 48 hours of the invitation email to be helpful?
Yes. No.
If No: Why not?

PURPOSE

3. Did you understand the objective of the SPIN-HAND program?
Yes. No.
If No: How could the objective be clarified?

4. Did you find the information provided in the SPIN-HAND program relevant?
Yes. No.
If No: How could the information provided be made more relevant for you or other scleroderma patients?

WORDS AND LANGUAGE

5. Did you find that the intervention used common, everyday language that was easy to understand?
Yes. No.
If No: Can you give an example of something or some word(s) that you did not understand?

6. Did you understand all the medical terms or, if not, were they clearly explained in the SPIN-HAND program?
Yes. No.
If No: Can you give an example of medical term(s) that you did not understand?

CONTENT, ORGANIZATION, NAVIGATION

7. Did you find that the SPIN-HAND program is broken down into manageable chunks or sections?
Yes. No.
If No: Which parts of the content weren’t broken down into manageable chunks or sections and how could we improve them?

8. Did you find the different pages or sections of the intervention were clearly indicated?
Yes. No.
If No: What section(s) could be more clearly labeled?

9. Did you find it easy to navigate through the intervention and to understand where to go next?
Yes. No.
If No: How could the different steps to navigate the intervention be more clearly explained?

10. Did you consult the "More info" tab (Scleroderma and your hands, FAQ, Patient stories)?
Yes. No.
If No: Why not?

11. Did you experience any technical difficulties while using the intervention?
Yes. No.
If Yes: What type of technical problems? Did you request assistance from the SPIN team? If you did, was the SPIN team able to help you resolve them?

12. Did you use the website tour?
Yes. No.
If Yes: Was it helpful to learn to navigate the website? Why or why not?

13. Did you use the "My bookmarks" feature?
Yes. No.
If Yes: Did you find it helpful for easily navigating to the pages you wanted? Why or why not?

VISUAL AIDS

14. Did the fact that the intervention was introduced by scleroderma experts and patients make the program more relatable?
Why or why not?

15. Did you understand how to correctly perform the exercises from watching the videos and listening to the audio instructions?
Yes. No.
If No: What would have helped you better understand how to correctly perform the exercises?

16. Did you take a look at the "Tips to avoid common mistakes" sections?
Yes. No.
If Yes: Did the pictures of common mistakes and written instructions help you to avoid performing wrong movements? Yes. No.
If No: Why didn't you use the section on common mistakes section?

17. Were you able to clearly understand the people speaking in the videos?
Yes. No.
If No: Why couldn't you understand the words in the videos? (e.g. too fast, too soft, mumbling, accent)?; Are there any videos in particular that were more difficult to understand than others? If yes, which one(s);

18. Did you look at the video transcripts?
Yes. No.
If Yes: Were the video transcripts helpful to you? Why or why not?

ACTIONABILITY (Routine, Goal-setting, motivation)

19. Did you set an exercise routine for yourself?
Yes. No.
If Yes: Did you find it easy to set an exercise routine for yourself using the materials in the SPIN-HAND program? Yes. No.
If No: How could the step-by-step approach be improved or better explained?

20. Did you find an exercise routine that fit your ability level and needs?
Yes. No.
If No: What made it hard for you to find an exercise routine that fit your ability level and needs? (e.g., levels not appropriate, time spent on exercises per day or per week not appropriate, other reason)

21. Did you set goals for yourself using the goal setting material?
Yes. No.
Why or why not?

22. Did you incorporate exercises into your planned routine and stick to it?
Yes. No.
If No: What were some obstacles you faced when trying to incorporate the exercises into your routine? How could the SPIN-HAND program have helped you to overcome these obstacles?

23. Did you use the option to share your goals with friends and family via email?
Yes. No.
If Yes: Did the option to share your goals with friends and family via email help you stick to your goals? Yes. No.
If No: What other motivational feature might have been more helpful?

24. Did you set email reminders for yourself?
Yes. No.
If Yes: Did having the option set email reminders for yourself help you incorporate the exercises into your routine? Yes. No.
If No: Did you use another type of reminder to do your exercises?

25. Did you use the feature to track your progress?
Yes. No.
If Yes: Did having the option to track your progress week after week encourage you to continue performing the exercises? Yes. No.
If No: Why not? Did you use any other way to track your progress? Is so, what did you do?

OVERALL APPRECIATION

26. How user-friendly on a 0-10 scale (0, being the worst and 10 being the best possible score) would you rate the SPIN-HAND program?

27. Would you recommend this program to someone with scleroderma?
Yes. No. If no, why?

28. What grade (on a 0-10 scale, 0 being the worst and 10 being the best possible score) would you give the program?
0 (worst) to 10 (best).

29. Is there anything you want to give us feedback about that was not included in this interview?
